# Changes in Child Nutrition in India: A Decomposition Approach

**DOI:** 10.3390/ijerph16101815

**Published:** 2019-05-22

**Authors:** Peng Nie, Anu Rammohan, Wencke Gwozdz, Alfonso Sousa-Poza

**Affiliations:** 1 School of Economics and Finance, Xi’an Jiaotong University, Xi’an 710061, China; 2 Department of Economics, University of Western Australia, Perth, WA 6009, Australia; anu.rammohan@uwa.edu.au; 3 Institute of Household Sciences, Justus-Liebig-University, 35390 Giessen, Germany; wencke.gwozdz@fb09.uni-giessen.de; 4 Department of Intercultural Communication and Management, Centre for Corporate Social Responsibility, Copenhagen Business School, 2000 Frederiksberg, Denmark; 5 Institute for Health Care & Public Management, University of Hohenheim, 70599 Stuttgart, Germany; alfonso.sousa-poza@uni-hohenheim.de

**Keywords:** child undernutrition, India, decomposition

## Abstract

Background: Improvements in child health are a key indicator of progress towards the third goal of the United Nations’ Sustainable Development Goals. Poor nutritional outcomes of Indian children are occurring in the context of high economic growth rates. The aim of this paper is to conduct a comprehensive analysis of the demographic and socio-economic factors contributing to changes in the nutritional status of children aged 0–5 years in India using data from the 2004–2005 and 2011–2012 Indian Human Development Survey. Methods: To identify how much the different socio-economic conditions of households contribute to the changes observed in stunting, underweight and the Composite Index of Anthropometric Failure (CIAF), we employ both linear and non-linear decompositions, as well as the unconditional quantile technique. Results: We find the incidence of stunting and underweight dropping by 7 and 6 percentage points, respectively. Much of this remarkable improvement is encountered in the Central and Western regions. A household’s economic situation, as well as maternal body mass index and education, account for much of the change in child nutrition. The same holds for CIAF in the non-linear decomposition. Although higher maternal autonomy is associated with a decrease in stunting and underweight, the contribution of maternal autonomy to improvements is relatively small. Conclusions: Household wealth consistently makes the largest contribution to improvements in undernutrition. Nevertheless, maternal autonomy and education also play a relatively important role.

## 1. Introduction

Improvements in child health are a key indicator of progress towards the third goal of the United Nations’ Sustainable Development Goals: A universal guarantee of a healthy life and well-being at all ages. Undernutrition puts children at a greater risk of disease vulnerability, also adversely affects their physical, cognitive, and mental development [1,2], may adversely impact productivity in later life [3] and increase economic inequality [4]. 

Globally, India performs poorly across standard child nutritional measures [5]. For child malnutrition, India ranked 114 out of 132 countries, just ahead of Afghanistan and Pakistan [6]. Data from India’s nationally representative National Family Health Survey (NFHS), from 1992–1993 to 2015–2016, paint a bleak picture of child nutrition. Although the prevalence of stunting among under-five children decreased from 52% to 38% and underweight declined from 53% to 36% between 1992 and 2016, prevalence is still alarmingly high [7]. In 2016, India had 62 million stunted children, accounting for 40% of the global share of stunting [7]. Large regional differences also exist with stunting over 46% and 48% in the states of Uttar Pradesh (India’s most populated state) and Bihar [8]. 

These poor nutritional outcomes of Indian children are occurring in the context of high economic growth rates, but with low levels of maternal autonomy. Furthermore, a large body of empirical research has linked greater maternal autonomy to better nutrition of children, particularly girls [9,10]. An improvement in maternal autonomy is expected to improve a mother’s ability to make decisions regarding her children’s health and nutrition; and a more autonomous mother is also likely to have greater access to resources, may lead to the adoption of healthy and diversified diets, improve the nutritional content of diets, contribute to better food hygiene and sanitation, and thereby reduce the risk of infection and disease. Since undernutrition is the outcome of insufficient food intake and repeated infectious diseases [11], it is imperative to understand the links between household-level socio-economic factors (and in particular the role of maternal autonomy) and the extent to which it manifests into poor nutritional outcomes for children. 

The aim of this paper, therefore, is to use the 2004–2005 and 2011–2012 data from the Indian Human Development Survey (IHDS) to conduct a comprehensive analysis of the demographic and socio-economic factors contributing to changes in the nutritional status (HAZ, WAZ and the Composite Index of Anthropometric Failure (CIAF)) of children aged 0–5 years in India. A special focus of our analysis is on the role that maternal autonomy plays in improving child undernutrition. Specifically, we address three key questions: (i) Have there been any changes in the nutritional status of children over the period 2004/2015–2011/2012? (ii) Do the changes differ across regions? (iii) What factors are associated with the changing nutritional outcomes of children, and, in particular, what role does maternal autonomy play? 

## 2. Prior studies

### 2.1. Socio-Economic Factors Associated with Child Undernutrition in India

A large body of research has used nationally representative secondary data to investigate the socio-economic factors associated with poor child nutrition in India. These studies find an increase in inequalities for vulnerable groups such as girls and lower socio-economic individuals [12,13,14,15,16]. Pathak and Singh [17] show that, over the period 1992–2006, the burden of undernutrition was disproportionately concentrated among poor children, with relatively better child nutrition in areas where households can access the Government funded Integrated Child Development Services (ICDS). Launched in 1975, the ICDS Program is a Government of India funded program aimed at improving the nutrition and health status of pre-school age children. In addition, Coffey [18] finds that state-level variation in neonatal mortality is associated with child heights. These findings are consistent with Subramanyam et al.’s [19] conclusion that over these 14 observation years, social disparities in undernutrition have either widened or stayed the same.

There is also empirical support for the role of maternal autonomy in influencing child nutrition [20,21]. In the Indian context, Shroff et al. [22] show that higher maternal autonomy (indicated by access to money and freedom to go to the market) is associated with a decreased risk of stunting among children aged below three years in the state of Andhra Pradesh. Similarly, Shroff et al. [23] find that maternal household decision-making autonomy is positively associated with weight-for-height (WHZ) and WAZ; while mobility autonomy is positively linked with HAZ when adjusting for birth weight among infants aged 3–5 months. Similarly, Imai et al. [24] show that maternal autonomy (measured by whether she is allowed to go to market without her husband’s permission) is positively associated with HAZ and WAZ; and Arulampalam et al. [25] demonstrate that maternal autonomy (a composite measure of decision-making, mobility and financial autonomy) has a significant positive impact on HAZ, but is not associated with WHZ. Interestingly, Imai et al. [24] further indicate that maternal autonomy is only associated with HAZ at the low end of the conditional distribution. Nevertheless, one recent study [25] shows that maternal decision-making autonomy is not associated with any of undernutrition outcomes when adjusting for maternal and household socio-economic factors. Thus, empirical evidence is mixed although the majority of studies support an association between child nutrition and maternal autonomy.

Another strand of literature focuses on the role of poor health infrastructure. For example, Paul et al. [26] attribute the poor nutritional outcomes among Indian children to weak health systems and a policy focus on children aged 3–6 years at the expense of those aged 0–2 years, although much of a child’s growth occurs in the early years. Spears [27] and Hammer and Spears [28] further explain poor child nutrition among Indian children in terms of sanitation, arguing that environmental threats from open defecation and exposure to fecal germs reduce nutrient absorption, while exposure to early life disease leads to undernutrition, stunting, and diarrhea. Using data from the NFHS, Spears [29] shows that open defecation remains exceptionally widespread in India and sanitation has not improved substantially despite rapid economic growth. Although these studies provide useful benchmarks for assessing the links between socio-economic characteristics and child nutrition, they are based on National Family and Health Survey (NFHS) and National Sample Survey (NSS) data sets that are over a decade old. 

### 2.2. Applying Decomposition Analyses to Explain the Socio-Economic Factors Underlying Child Undernutrition in India

The three major decomposition techniques, including Blinder-Oaxaca (BO) linear decomposition, nonlinear decomposition, and quantile-based decomposition, have been used in previous studies in India to analyze the gap in child undernutrition/health between certain groups (such as poor/non-poor, Muslims/Hindu, rural/urban). BO decomposition is used to decompose differences in a continuous variable (e.g., child undernutrition outcome) into a part attributable to differences in characteristics (explained part or endowments part) and a part attributable to coefficients (unexplained part or effects part). Nonlinear decomposition, in essence, employs an extension of the BO decomposition for binary variables (e.g., underweight or stunting). BO decomposition is a mean-based approach, yet covariate and coefficient contributions may differ at different parts of the distribution of undernutrition. Quantile-based decomposition is thus used to explore the contributions of covariates at different quantiles of the outcome distribution. For example, Bhalotra et al. [30] apply a non-linear decomposition technique [31] to three waves of the NFHS (1992/1993, 1998/1999, and 2005/2006) to measure the Hindu-Muslim gap in under-five child undernutrition. They show that the 29% difference in stunting between these two groups is mainly attributable to maternal education, maternal age at parturition, and child’s birth year, while the 20% gap in wasting is primarily explainable by maternal education and state of residence. Similarly, Kumar and Singh [32] apply the BO decomposition method to 2005–2006 NFHS data to measure the gap in under-five child undernutrition between poor and non-poor households in urban India. They identify the main contributing factors as underutilization of health care services, poor maternal body mass index (BMI), and low levels of parental education among impoverished urbanists. 

In a regression-based decomposition of the same datasets to assess (concentration index-based) inequalities in under-five child mortality and undernutrition outcomes, Chalasani [33] identifies wealth and mother’s education as the two largest contributors to severe stunting and severe underweight inequality over the 1992/1993–2005/2006 period. These results are supported by Kumar and Kumari [34], who use BO decomposition to show that household economic status (wealth score) and parental education are the most significant contributors to the rural-urban gap in childhood undernutrition in India (measured using z-scores of weight-for-age). Similarly, using the 2005–2006 NFHS data, Mazumdar [35] identifies household wealth and mother’s education as the two largest contributors to inequality in child undernutrition in explaining the child undernutrition inequalities. Van de Poel and Speybroeck [36], in their earlier BO decomposition of 1998–1999 IDHS data, attribute the observed child undernutrition gap among scheduled castes and scheduled tribes primarily to their lower wealth, education level, and use of health care services. Cavatorta et al. [37] use Machado and Mata’s conditional quantile decomposition approach [38] to show that the surprisingly modest height-for-age disparities across six Indian states can be explained by covariate differences in endowment effects.

Summing up, previous analyses of undernutrition changes in pre-school age Indian children point to household economic status (particularly wealth) and maternal education as the two most important contributors. With few exceptions, however, this research predominantly uses BO decomposition, which can provide misleading estimates when the outcome variable is binary and explanatory variables differ substantially across groups [39]. To the best of our knowledge, only two studies analyze child undernutrition using non-linear decomposition: Bhalotra et al. [30], who use the Fairlie method to identify Hindu-Muslim disparities in under-five child mortality and undernutrition, and Cavatorta et al. [37], who employ Machado and Mata’s conditional quantile decomposition technique [38] to explore the relative contributions of covariates and coefficients over the entire height-for-age distribution. Moreover, we are not aware of previous studies using the Fairlie non-linear decomposition to examine anthropometric failure differences between groups or over time, and the Machado and Mata’s method is not extendable to a detailed decomposition for each determinant. We address both these research gaps and conduct a comprehensive analysis of the demographic and socio-economic factors contributing to changes in the nutritional status (HAZ, WAZ and CIAF) of children aged 0–5 years in India using the IHDS data for 2004–2005 and 2011–2012. 

## 3. Materials and Methods

### 3.1. Data

The data for this analysis are taken from the IHDS 2004–2005 and 2011–2012, a collaborative research program between researchers from the National Council of Applied Economic Research, New Delhi, and the University of Maryland. This nationally representative multi-topic survey was administered to households in 1503 villages and 971 urban neighborhoods across India and the sample includes 384 districts out of a total of 593 identified in 2001 census. Villages and urban blocks (comprising of 150–200 households) form the primary sampling unit (PSU) from which the households are selected [40]. Urban and rural PSUs are selected using a different design. Specifically, to draw a random sample of urban households, all urban areas in a state are listed in the order of their size with the number of blocks drawn from each urban area allocated based on probability proportional to size [40]. When the numbers of blocks for each urban area are fixed, the enumeration blocks are then selected randomly with the assistance from Registrar General of India. Drawing on these Census Enumeration Blocks of about 150–200 households, a complete household listing is conducted and a household sample of 15 households is selected within each block. For sampling purposes, some smaller states are merged with nearby larger states. Nevertheless, the rural sample encompasses about half the households that are interviewed initially by NCAER in 1993–1994 in a survey titled Human Development Profile of India (HDPI) and the other half of the samples are drawn from both districts surveyed in HDPI as well as from the districts located in the states and union territories not covered in HDPI [40]. The first phase, IHDS-I (2004–2005), comprised two one-hour interviews with each household on topics such as health status, education, employment, economic status, marriage, fertility, gender relations, and social capital. The second phase, IHDS-II, was conducted between 2011–2012. A detailed description of sampling design and data quality is available in Reference [40]. All individual- and household-level data are available for public use [41].

Our sample is restricted to those households with children born in the five years prior to the survey, where information was available on all our variables of interest. Because data on certain outcome variables of interest are limited, our final pooled sample contains 6445 observations for stunting, 7634 observations for underweight, and 5693 observations for the CIAF. 

### 3.2. Study Variables

#### 3.2.1. Dependent Variables

In keeping with the World Health Organization’s reference standards [42], we measure children’s nutritional outcomes conventionally using z-scores of height-for-age (HAZ) and weight for age (WAZ). According to Waterlow et al. [43], the height-for-age z-score, expressed in standard deviations from the reference population mean, is a good indicator of nutritional status. Whereas HAZ measures long-term nutrition by showing the cumulative effects of growth deficiency (often associated with chronic insufficient food intake, frequent infections, sustained incorrect feeding practices, and/or low socio-economic family status), WAZ reflects both acute and chronic undernutrition, making it a better single indicator of childhood undernutrition [44]. 

Children with z-score values below −2 (below −3) of the reference population are considered undernourished (severely undernourished) [42]. However, because these conventional undernutrition measures reflect different aspects of anthropometric failure, they cannot individually determine the overall prevalence of child undernutrition in a population, and may underestimate the true extent of undernutrition, primarily due to the overlapping of children into multiple categories of anthropometric failure [45,46,47,48]. For instance, underweight cannot identify children who are suffering from underweight combined with stunting and/or wasting [46,47]. We address this shortcoming using Svedberg’s [48] CIAF, an aggregated single anthropometric proxy for the overall estimation of malnourished children. In our analysis, we combine Nandy et al.’s [45] Group Y, underweight only, with six of Svedberg’s groups [48]: Group A, no failure; Group B, wasting only; Group C, wasting and underweight; Group D, wasting, stunting, and underweight; Group E, stunting and underweight; and Group F, stunting only (see Table A1 for a detailed classification). CIAF is thus a binary variable for which 0 indicates no failure, and 1 signals one or more anthropometric failures.

#### 3.2.2. Explanatory Variables

Maternal characteristics. We control for mother’s education using four categories: No education, primary, secondary, tertiary and above. As a proxy for mother’s health, we include her BMI measured in kg/m^2^ (categorizing into three groups: Underweight for BMI < 18.5, normal for 18.5 ≤ BMI ≤ 24.9 and overweight/obesity for BMI ≥ 25). 

Economic characteristics. A household’s economic status is measured using the household wealth index, which is a categorical variable divided into five population quintiles from the poorest 20% to the wealthiest 20% of households [49]. This index is calculated with Principle Component Analyses (PCA) using 33 dichotomous items measuring household ownership of assets and housing quality. Relative to income and consumption, this measure of wealth is less volatile and thus arguably a better long-run measure of household economic status. 

Maternal autonomy. A key advantage of our dataset is the rich array of attitudinal questions that are available on married women’s decision-making authority in the household. Autonomy is regarded as a multidimensional construct, encompassing dimensions such as the ability to make purchases, control over resources, decision-making autonomy both relating to own health care or child’s medical needs [50]. We therefore categorize maternal autonomy into:

- Decision-making autonomy: A female respondent (child’s mother) is assumed to have decision-making autonomy if she was involved in decision-making either on her own or in conjunction with another household member on: (i) What is to be cooked, (ii) making expensive purchases, (iii) the number of children to have, and (iv) children’s medical needs (decide what to do when a child falls sick).
- Mobility autonomy: The child’s mother is assumed to have mobility autonomy if she can go on her own: (i) To visit relatives/friends, (ii) to the local health center, and (iii) to the local grocery store.

These individual responses are coded as binary indicators which we use to construct two factors: Maternal autonomy in decision-making and mobility using PCA.

Hygiene characteristics. We include three binary variables that capture the level of sanitation and hygiene practiced in the household: Drinking water source (1 if the household’s drinking water is piped or supplied by tube well or hand pump, 0 otherwise), access to a flushing toilet (1 = yes, 0 = no), and hand-washing behavior (1 = yes, 0 = no).

Regional characteristics. The 24 Indian states are classified into six regions as per the regional definitions used in the IHDS data (see Table A2). These are: North (comprising of the states of Jammu and Kashmir, Himachal Pradesh, Punjab, Chandigarh, Uttaranchal, Uttar Pradesh, Haryana and Delhi); Central (Chhattisgarh and Madhya Pradesh); East (Bihar, West Bengal, Jharkhand, Odisha); South (Andhra Pradesh, Karnataka, Kerala, Tamil Nadu and Pondicherry); North East (Sikkim, Arunachal Pradesh, Nagaland, Manipur, Mizoram, Tripura, Meghalaya and Assam); and West (Rajasthan, Goa, Maharashtra, Daman and Diu, Dadar and Nagar Haveli and Gujarat). The descriptive analysis of regional changes in child nutrition are based on these six regions. 

Other characteristics. Our specifications also include controls for child’s age (in years) and gender (1 = male and 0 = female), father’s education levels (a categorical variable, 1 = no education, 2 = primary, 3 = secondary and 4 = tertiary and above), religion (a categorical variable, 1 = Hindu, 2 = Muslim and 3 = others), caste (a categorical variables, 1 = other, 2 = other backward and 3 = scheduled caste/tribe) and a binary variable for rural residence (1 = rural and 0 = urban).

### 3.3. Estimation Procedure

*Blinder-Oaxaca (BO) decomposition*. We use BO decomposition to explain changes in the nutritional measures HAZ and WAZ as a function of selected explanatory factors. The BO decomposition quantifies the distribution differences of factors that explain the average gap, and also identifies differences in these factors’ effects [51]. The total difference in mean z-scores of our three measures of child undernutrition can be decomposed as follows:

(1) $${\bar{Y}}^{2011/12}-{\bar{Y}}^{2004/05}=\left({\bar{X}}^{2011/12}-{\bar{X}}^{2004/05}\right){\hat{\beta }}^{2011/12}+{\bar{X}}^{2004/05}\left({\hat{\beta }}^{2011/12}-{\hat{\beta }}^{2004/05}\right)$$

where ${\bar{X}}^{i}$ is a vector of the averaged values of the independent variables and ${\hat{\beta }}^{i}$ is a vector of the coefficient estimates for wave *i* (here, *i* = 2004/2005, 2011/2012). 

*Re-centred influence function regression (RIFR) decomposition*. Because covariate and coefficient contributions may differ between the median and tails of the childhood undernutrition distribution, we use RIFR decomposition [52] to investigate the contributions of demographic and socio-economic characteristics at different quantiles of the unconditional marginal distribution. The RIFR method involves a two-step procedure: First, we calculate an influence function (IF) at each quantile τ of the distribution of the outcome variable (z-score of child undernutrition), as follows:

(2) $$RIF\left(zscore;q_{\tau }\right)=q_{\tau }+(\tau -1\left[zscore\leq q_{\tau }\right])/f_{zscore}\left(q_{\tau }\right)$$

where $q_{\tau }\;$represents the unconditional τth quantile of the *z*-score, $f_{zscore}\left(q_{\tau }\right)$ is the unconditional density of the *z*-score at the τth quantile, and $1\left[zscore\leq q_{\tau }\right]$ is an indicator function for whether the outcome variable is smaller or equal to the τth quantile. For each quantile, the coefficient on *X* for waves 2004/2005 and 2011/2012 are then estimated by regressing the RIF on *X*:

(3) $$q_{wave,\;\tau }=E_{X}\left[E\left[\hat{RIF}\left(zscore;q_{wave,\;\tau }\right)|X_{wave}\right]\right]=E\left[X_{wave}\right]{\hat{\theta }}_{wave,\tau }$$

where $q_{wave,\;\tau }$ is the unconditional τth quantile of the *z*-score for wave 2004/05 and 2011/12, respectively. ${\hat{\theta }}_{wave,\tau }$ is the coefficient of the unconditional quantile regression, which captures the marginal effect of a change in the distribution of *X* on the unconditional quantile of the *z*-score. 

In the second step, we employ the BO decomposition strategy at different quantiles (25%, 50%, and 75%) calculated by the RIFR:

(4) $${\hat{\Delta }}_{zscore}^{\tau }=\left[\hat{RIF}\left(zscore_{2011/12};q_{2011/12,\;\tau }\right)\right]-\left[\hat{RIF}\left(zscore_{2004/05};q_{2004/05,\;\tau }\right)\right]$$

(5) $${\hat{\Delta }}_{zscore}^{\tau }=\left({\bar{X}}_{2011/12}-{\bar{X}}_{2004/05}\right){\hat{\theta }}_{2011/12,\tau }+{\bar{X}}_{2004/05}\left({\hat{\theta }}_{2011/12,\tau }-{\hat{\theta }}_{2004/05,\tau }\right)$$

Both the explained and unexplained parts are then decomposed into the contributions of each covariate at the τth quantile in Equation (5), which is in effect analogous to the BO decomposition in Equation (1). 

*Fairlie’s (1999) non-linear decomposition.* Applying standard BO decomposition to a linear probability model provides misleading estimates for binary dependent variables, particularly if the group differences for an influential independent variable are relatively large [39]. It is therefore preferable to apply a relatively straightforward simulation technique for non-linear decomposition. Accordingly, we estimate the contributions of socio-economic and demographic factors to identified differences in our key undernutrition indicators by employing a non-linear decomposition approach for binary dependent variables. Stunting, underweight, and CIAF are the dependent variables, so the decomposition for the non-linear equation, $Y=F\left(X\hat{\beta }\right)$, can be expressed as:

(6) $$\begin{array}{c} {\bar{Y}}^{2011/12}-{\bar{Y}}^{2004/05}\\ \;=(\sum_{i=1}^{N^{2011/12}}\frac{F\left(X_{i}^{2011/12}{\hat{\beta }}^{2004/05}\right)}{N^{2011/12}}\\ \;-\sum_{i=1}^{N^{2004/05}}\frac{F\left(X_{i}^{2004/05}{\hat{\beta }}^{2004/05}\right)}{N^{2004/05}})\\ \;-\sum_{i=1}^{N^{2011/12}}\frac{F\left(X_{i}^{2011/12}{\hat{\beta }}^{2011/12}\right)}{N^{2011/12}}\\ \;-\sum_{i=1}^{N^{2011/12}}\frac{F\left(X_{i}^{2011/12}{\hat{\beta }}^{2004/05}\right)}{N^{2011/12}}) \end{array}$$

where $N^{j}$ denotes the sample size of each wave (*j* = 2004/2005, 2011/2012). The function F(.) represents a probit model. Two aspects are worth noting: First, the BO decomposition in Equation (1) is a special case of Equation (6) where $F\left(X_{i}\beta \right)=X_{i}\beta$. Second, in Equations (1) and (6), the first (explained) term on the right indicates the contribution resulting from a difference in the distribution of the determinant of *X*, and the second (unexplained) term refers to the part attributable to a difference in the effect of the determinants. Equally noteworthy, the second term captures all the potential effects of differences in unobservables [39]. In keeping with previous research using decomposition, we focus on the explained terms and their disaggregated contribution for individual covariates, which result primarily from the difficulty of interpreting the unexplained part [53]. The contribution of a variable is given by the average change in the function if that variable is changed while all other variables remain the same. For severe childhood undernutrition in terms of *HAZ* and *WAZ*, we use the same specification as in Equation (6).

One potential concern related to Fairlie’s sequential decomposition is path dependence, the possibility that changing the order of variables in the decomposition may produce different results [39,54]. We therefore test the sensitivity of decomposition estimates to variable re-ordering by randomizing their order in the decomposition [39] using 1000 replications, the minimum number recommended for most applications [39]. As a robustness check, we also perform an analysis using 5000 replications. These results are not reported here but are available on request.

When reporting the decomposition results for these three decomposition methods, we categorize the disaggregated contributions of the determinants in the explained part into five main dimensions depicted above, namely: Maternal autonomy, maternal characteristics, household economic status, hygiene, and other.

## 4. Results

### 4.1. Descriptive Statistics

**Figure 1:**
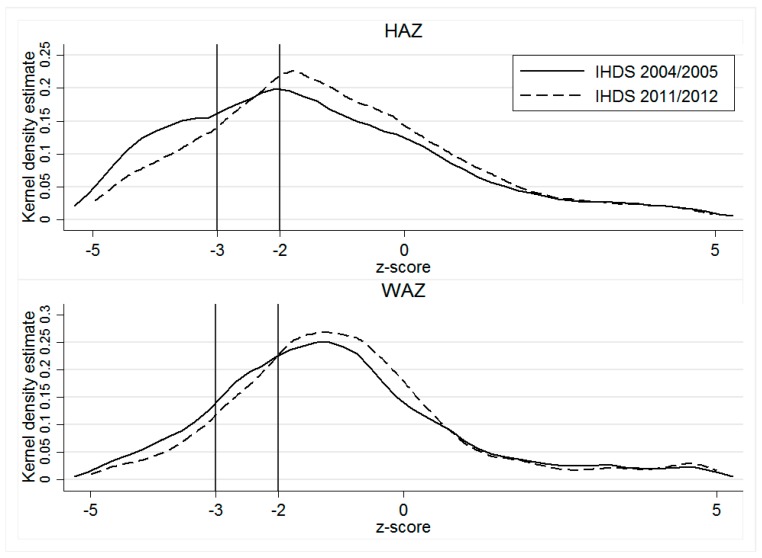
Kernel density estimates for *HAZ* and *WAZ* by year. Notes: Kolmogrov–Smirnov test *p*-value: *HAZ*: Combined K–S 0.098; *p*-value = 0.000; *WAZ*: Combined K–S 0.062; *p*-value = 0.000.

As Table 1 shows, stunting, underweight, and anthropometric failure improved significantly over the 2004–2012 period (a 43% to 36% decline in stunting, a 33% to 28% decline in underweight, and a 58% to 50% decline in CIAF). Severe stunting declined from 25% to 18% and severe underweight from 14% to 10%. In keeping with previous research, the CIAF indicates a much higher prevalence of undernutrition than the individual measures [45,46,47]. Nevertheless, the improvements within the 7-year timeframe of our analysis are remarkable. These significant improvements in malnutrition are also depicted in the kernel densities of the z-scores stratified by year in Figure 1. Together with this significant improvement in malnutrition we also observe a large improvement in the economic status of households (household wealth): 6 percentage points increase for the rich and 9 percentage points for the richest households. We also note that the majority of items depicting maternal autonomy have improved. 

Regional differences in child nutrition outcomes are presented in Table 2. The main results in Table 2 are: (i) Large and statistically significant improvements in all nutrition measures in Central; (ii) Although not as large as in Central, all measures improved in the West; (iii) No improvements at all in East and South; (iv) Stunting, underweight and severe underweight improved slightly in the North, although severe stunting has actually gotten worse. Our composite measure has also gotten worse in this region; and finally, (v) in the Northwest, there are no significant changes in our conventional measures, yet a dramatic improvement in our composite measure.

### 4.2. Explaining the Differences in Nutritional Outcomes

#### 4.2.1. BO Decomposition Estimates

The results of the conventional BO decomposition for the HAZ and WAZ z-scores are reported in Table 3. For HAZ and WAZ, we observe that the contributions of the explained part are 9% and 38%, respectively. As regards the separate contributions to the explained part, household wealth is the most important contributor to the improvement in the average values of HAZ (21%) and WAZ (25%). Maternal mobility autonomy accounts for approximately 4% of the improvements in the average values of HAZ and WAZ. Interestingly, we do not find any associations of maternal decision-making autonomy with nutritional outcomes. In order to assess whether the influence of maternal autonomy is associated with a mother’s age, we also split our full sample into two groups (mothers aged ≤25 and mothers aged >25) and rerun the estimates. Results (presented in Table A3) indicate that, especially for younger mothers aged ≤25, mobility autonomy is an important contributor explaining 12% (6%) of the difference in HAZ (WAZ). Maternal autonomy matters less for older mothers. We also find that regions (taking East as the reference group as there are no significant improvements of all child undernutrition indicators in this region), in particular, North (−13%) and South (8%), play a relatively important role in explaining changes in HAZ. Note that the negative contribution of North actually implies that this region should actually decrease the national difference in HAZ. We observe no significant contributions in the case of WAZ.

#### 4.2.2. RIFR Decomposition Estimates

The results of the RIFR decomposition are presented in Table 4. We observe large differences in malnutrition between the two surveys, particularly at the bottom end of the distributions, and insignificant at the upper end (75th quantile). The decomposition analysis produces three noteworthy findings: First, household wealth makes the largest contribution to the overall explained part for both HAZ and WAZ (panels A and B, respectively), especially at the lower parts of the distribution (for HAZ, 25th quantile: 25%, 50th quantile: 37%; for WAZ, 25th quantile: 22%, 50th quantile: 61%). Second, in the lowest part of the distribution (25th quantile) of HAZ (Panel A), about 4% of the improvements can be explained with maternal characteristics of BMI and education and about 5% and 6% in the median quantile respectively. With regards to WAZ (Panel B), the contribution of maternal characteristics to the explained component becomes even larger, ranging from 6% at 25th quantile to 16% at the median. Third, mobility autonomy’s contribution to the improvement in HAZ ranges from 4% to 5% at the 25th and 50th quantiles, respectively (panel A). Additionally, we also find that regions (North and South) also make a relatively large contribution to the overall explained part for HAZ, though the relative contributions of North and South are much larger at the median level than these at the 25th quantile (North: 14% versus -6%; South: 15% versus 5%). However, we only observe a larger contribution of Southern region (10%) to the overall explained part for WAZ at the median level. 

#### 4.2.3. Fairlie Nonlinear Decomposition Estimates

The results of Fairlie non-linear decomposition are presented in Table 5. As can be seen, the contributions of the explained part vary substantially for the different measures of child undernutrition: 12% for stunting, 8% for underweight, and 40% for the CIAF. For the individual contribution of each dimension in the explained part, household economic status (household wealth) uniformly explains the largest proportion of improvements in stunting, underweight, and anthropometric failure, with contributions of 20%, 24%, and 19%, respectively. Likewise, maternal characteristics (including maternal BMI and education) explain 3% of stunting, 5% of underweight, 7% of anthropometric failure. Maternal mobility autonomy accounts for about 3% of the improvement in all measures. We also find that regions (especially South) play an important role in accounting for the improvements in stunting and CIAF (stunting: 7%; CIAF: 11%). In addition, the Southern region also contributes to around 3% of the improvement of underweight.

We also examine severe forms of stunting and underweight (z-score < −3). In Table 6 we note that, once again, household wealth is the most important contributor to improvements in severe childhood undernutrition, accounting for 22% and 20% of the improvement in stunting and underweight, respectively. Maternal mobility autonomy explains around 3% of the variation in both severe underweight and stunting. In addition, the Northern region makes a moderate contribution (9%) to the improvements in severe underweight. The Southern region contributes approximately 4% of the change in severe stunting.

Taken together, the results for the BO, non-linear, and RIFR decompositions suggest that household economic status consistently makes the largest contribution to the improvements in child undernutrition, especially at the lower ends of the distributions. The same holds for CIAF in the non-linear decomposition. Maternal characteristics of BMI and education also make a relatively important contribution. Furthermore, regions, in particular, North and South, account for improvements in child undernutrition. The RIFR quantile-based decomposition further indicates that North and South make relatively larger contributions to the total explained part in HAZ, especially at the median level.

## 5. Discussion

The poor nutritional outcomes for children in India, coupled with the high economic growth rates in recent decades, have been the subject of much research. We contribute to this research by analyzing data from phases I (2004–2005) and II (2011–2012) of the nationally representative Indian Human Development Study in order to provide a comprehensive econometric analysis of the key demographic and socio-economic factors associated with the changes in undernutrition of Indian children under five years. Our study makes a number of important contributions to the literature. First, our analysis provides a comprehensive empirical analysis of the nutritional status of India’s children aged 0–5 years, focusing on changes over the period 2004–2012, using data from the nationally representative Indian Human Development Survey (IHDS). Second, we examine regional differences in child nutrition, with a focus on the regional changes in malnutrition between 2004–2012. Third, we analyze the role of different dimensions of maternal autonomy on child nutrition. Fourth, we identify how much the different dimensions of maternal autonomy as well as the general socio-economic conditions of households contribute to the changes observed in child undernutrition. To identify each dimension’s contribution, we employ both linear Blinder-Oaxaca (BO) and non-linear decompositions [55], as well as the unconditional quantile technique developed by Firpo et al. [52]. 

### 5.1. Key Findings

Our analysis finds a pronounced improvement in stunting, underweight, and overall anthropometric failure, as well as in severe undernutrition, especially severe stunting. Considering the relative short time period under analysis, these improvements are impressive, with the incidence of stunting and underweight dropping by 7 and 6 percentage points, respectively. However, the CIAF scores reveal a much higher prevalence of child undernutrition, suggesting that conventional undernutrition indicators like stunting, and underweight may underestimate its actual extent. There are, however, large regional differences in child nutritional outcomes. The marked improvements in outcomes are particularly evident in the Central region and in the West, with smaller declines observed in the North. Very little improvement can be observed in the South and East. The positive development in the Central region may be attributable to having a nutrition mission and state-level interventions in maternal nutrition in the Central states of Chhattisgarh and Madhya Pradesh. For example, in the state of the central state of Chhattisgarh, 83% of the beneficiaries with children aged 6–35 months received supplementary feeding under ICDS, with the figure being 60% for those with children under 36–71 months [56]. In terms of growth monitoring, 88% of the Anganwadi Centers (AWCs) under the ICDS program had access to functional weighing scale, 86% of the Anganwadi workers (AWWs) had correct knowledge of intake of food by pregnant women and in terms of health service delivery personnel, 100% of the ASHAs selected were in post. In contrast in Uttar Pradesh in the North, only 23% (23) of the beneficiaries with children in the 6–35 month (36–71 months) age-group received supplementary feeding under ICDS; only 50% of the AWCs had functioning baby weighing scales and only 85% of the ASHAs had been appointed.

All three decomposition techniques (BO, non-linear, and RIFR) indicate that household economic status (indicated by household wealth) consistently makes the largest contribution to improvements in undernutrition. These results are in line with the descriptive statistics where we observe a large decline in the proportion of children in the poorest household wealth, and echo previous findings by Chalasani [33], Van de Poel and Speybroeck [36], and Mazumdar [35]. Nevertheless, maternal education and BMI also play a relatively important role. Our unconditional-quantile decomposition also confirms that household economic status and maternal characteristics primarily affect the lower ends of the distribution (25% and 50% quantiles) of HAZ and WAZ. This may imply stronger impacts of household wealth, maternal BMI and education on under-five children with lower HAZ and WAZ. 

In addition, BO decomposition indicates that regions, especially North and South, also play a relatively important role in accounting for the overall explained part in HAZ. Results from unconditional quantile decomposition further reveal that both regions make a significant contribution to the overall explained part for HAZ, especially at the median level of the distribution. Nonlinear decomposition results demonstrate that both regions also make relatively larger contributions to the improvements in stunting. One possible explanation is the economic growth observed over this period, which could have led to large declines in stunting particularly in the Northern states.

Maternal mobility autonomy makes a relatively small contribution to the improvements in both HAZ and WAZ, ranging from 3%–5%. This finding might be attributable to the possibility that those mothers with higher mobility autonomy are more likely to attend postnatal check-ups, monitor adequate child growth and obtain professional advice on health care [23]. Interestingly, the contribution of maternal decision-making autonomy is negligible, which is also echoed by Rajaram et al. [57] using the 2005/2006 NFHS. One possible explanation for this finding is that, even though greater maternal autonomy will improve child nutritional status, this is based on the assumption that women are well-educated, aware of best child-care practices and care about their children [25]. Thus, without supporting infrastructure and policies, maternal autonomy may come at the expense of less time for child care, particularly for those in the labor market [25]. Our results also provide evidence that maternal autonomy is particularly important for younger mothers. The United National Children’s Fund’s (UNICEF) conceptual framework for undernutrition defines basic, underlying, and immediate causes of child undernutrition (including individual, household, and environmental factors) [58]. Generally, our results find that household wealth, maternal BMI, education and autonomy play a key role in the improvements of Indian child undernutrition, which are well in line with the basic causes of child undernutrition in the UNICEF conceptual framework of nutrition; in particular, household access to adequate financial, human, physical and social capital [58].

### 5.2. Limitations

Some limitations should be taken into account: First, omitted variables may potentially bias the estimates. For instance, better indicators of maternal health, campaigns/programs/other structural changes, the availability of public health services and environmental factors that may have influenced children’s nutritional status were not included in the model due to data availability. Second, all three decomposition approaches (mean-based BO, Fairlie’s nonlinear and unconditional quantile-base) decompose a difference without assessing causality. Finally, as we have observed in this study, child undernutrition shows a large variation across geographical areas of India. Due to data limitations (most notably small sample sizes), we are unable to explore what drives these spatial heterogeneities of undernutrition at a state or district level [7].

### 5.3. Future Research Directions

The limitations of this study point to several future research directions. First, more focused analyses that have detailed information on maternal health, campaigns/programs/other structural changes, the availability of public health services and environmental factors could provide additional insights into the causes of undernutrition. Second, there is a dearth of research on the causal relationship between maternal autonomy and child undernutrition in India. Having appropriate data and a convincing identification strategy is a challenging endeavor for future research. Third, as we have observed, given the huge geographical/regional differences in child undernutrition in India, it is important to assess spatial-temporal heterogeneities across states or even districts. Finally, future research is needed to track the variations in child undernutrition and explore the underlying drivers over a longer time period.

## 6. Conclusions

As highlighted by the 2013 Lancet Maternal and Child Nutrition Series (MCNS) [59], the new sustainable development agenda should prioritize all forms of undernutrition, with a special emphasis on nutrition-specific interventions and programs (e.g., addressing the immediate determinants of child undernutrition), nutrition-sensitive programs and approaches (e.g., addressing the underlying determinants of child undernutrition), and building an enabling environment (e.g., addressing the basic determinants of child undernutrition). Our research has highlighted some important factors (household wealth, maternal BMI, autonomy, and education) associated with changes in undernutrition among Indian children. Our analysis also shows, however, that the impressive improvements in undernutrition among children under five in India go well beyond the improvements one would expect based on the developments of these determinants. This observation may well point to the effectiveness of public programs such as the ICDS [60], which has experienced a funding increase from US$35 million in 1990 to US$170 million in 2000, and a 2005 Indian government decision to give high priority to its expansion. Several other national and regional nutrition and education programs, including the National Midday Meal Scheme, the National Rural Health Mission, the Comprehensive Rural Health Project, the Integrated Nutrition and Health Program, and the Public Distribution System may have contributed to this improvement in undernutrition. 

## Figures and Tables

**Table 1:** Descriptive statistics of nutritional outcomes and covariates, IHDS 2004/2005–2011/2012.

Variable	2004/2005	2011/2012	Mean/Percentage Change
Z-score indicators			
Height-for-age z-score (*HAZ*)	−1.3931	−1.1682	0.2249 ***
Weight-for-age z-score (*WAZ*)	−1.0870	−0.9484	0.1385 ***
Nutritional status			
Stunting (*HAZ* < −2)	0.4294	0.3577	−0.0717 ***
Underweight (*WAZ* < −2)	0.3332	0.2746	−0.0586 ***
Severe stunting (*HAZ* < −3)	0.2534	0.1777	−0.0756 ***
Severe underweight (*WAZ* < −3)	0.1389	0.1020	−0.0369 ***
*CIAF*	0.5793	0.4969	−0.0824 ***
Maternal education			
No education	0.5689	0.4879	−0.0811 ***
Primary	0.0860	0.1055	0.0196 ***
Secondary	0.2899	0.3668	0.0769 ***
Tertiary and above	0.0552	0.0398	−0.0154 ***
Maternal BMI (kg/m^2^)			
<18.5 (underweight)	0.2778	0.3008	0.0230 ***
18.5−24.9 (normal)	0.6345	0.5770	−0.0574 ***
≥25 (overweight/obesity)	0.0877	0.1222	0.0344 ***
Economic situation (household wealth)			
Poorest	0.3522	0.1963	−0.1558 ***
Poor	0.2347	0.2473	0.0127
Middle	0.1944	0.1912	−0.0032
Rich	0.1175	0.1783	0.0609 ***
Richest	0.1013	0.1868	0.0855 ***
Maternal autonomy			
Can decide what is to be cooked	0.9566	0.9444	−0.0123 **
Can decide on expensive purchases	0.7451	0.7979	0.0528 ***
Can decide number of children	0.8321	0.9144	0.0823 ***
Can decide on children’s medical needs	0.8672	0.8969	0.0298 ***
Allowed to the home of relatives/friends alone	0.7010	0.7371	0.0361 ***
Allowed to local health center alone	0.6715	0.6733	0.0018
Allowed to kirana shop (grocery store) alone	0.7341	0.7570	0.0230 **
Factor 1 (decision-making autonomy)	0.8759	0.9284	0.0525 ***
Factor 2 (mobility autonomy)	0.7795	0.8159	0.0364 ***
Hygiene			
Water source	0.8339	0.8454	0.0115
Flushing toilet	0.1754	0.2544	0.0790 ***
Hand washing	0.9952	0.9948	−0.0004
Other controls			
Child’s age (in years)			
0 ≤ age ≤ 1	0.2682	0.2719	0.0037
1 < age ≤ 3	0.2244	0.2154	−0.0089
3 < age < 5	0.5074	0.5127	0.0052
Child gender			
Male (1 = male, 0 = female)	0.5180	0.5206	0.0026
Father’s education			
No education	0.3789	0.3103	−0.0685 ***
Primary	0.0973	0.1323	0.0350 ***
Secondary	0.4411	0.4881	0.0470 ***
Tertiary and above	0.0827	0.0693	−0.0134 **
Caste			
Other	0.2339	0.1961	−0.0379 ***
Other backward	0.4116	0.4123	0.0007
Scheduled caste/tribe	0.3544	0.3916	0.0372 ***
Religion			
Hindu	0.7696	0.7720	0.0024
Muslim	0.1646	0.1893	0.0246 ***
Other	0.0658	0.0387	−0.0271 ***
Rural	0.6420	0.7221	0.0801 ***

Notes: Mean values are reported for variables in the IHDS 2004/2005 and 2011/2012. Observations for HAZ (stunting/severe stunting) are 3272 in 2004/2005 and 3173 in 2011/2012, respectively. Observations for WAZ (underweight/severe underweight) are 3967 in 2004/2005 and 3667 in 2011/2012, respectively. Observations for CIAF are 2807 in 2004/2005 and 2886 in 2010/2011, respectively. The observations for other independent variables are the same as those of WAZ. The significance of the changes is based on independent t-tests. * *p* ≤ 0.05, ** *p* ≤ 0.01, *** *p* ≤ 0.001.

**Table 2:** Descriptive statistics (z-score indicators and nutritional status changes between IHDS 2004/2005 and 2011/2012).

Variable	Northwest	North	South	Central	East	West
Z-score indicators						
Height-for-age z-score	0.7477 *	0.1469	−0.0811	1.0993 ***	−0.0132	0.1830
Weight-for-age z-score	0.4178	0.0192	−0.2132 **	0.6556 ***	0.0161	0.3012 ***
Nutritional status						
Stunting	−0.0936	−0.0404 *	−0.0319	−0.2457 ***	−0.0186	−0.0751 ***
Underweight	−0.0818	−0.0379 **	0.0223	−0.1754 ***	−0.0200	−0.0956 ***
Severe stunting	−0.0954	0.0637 ***	−0.0284	−0.2216 ***	−0.0364	−0.0565 **
Severe underweight	0.0387	−0.0421 ***	0.0068	−0.1095 ***	−0.0010	−0.0441 ***
*CIAF*	−0.2602 ***	0.0459 *	−0.0340	−0.1763 ***	−0.0166	−0.1246 ***

Notes: Mean/percentage values are reported for key variables in the IHDS 2004/2005 and 2011/2012. The significance of the changes is based on independent t-tests. * *p* ≤ 0.05, ** *p* ≤ 0.01, *** *p* ≤ 0.001.

**Table 3:** Blinder-Oaxaca (BO) decomposition of socio-economic differences in undernutrition among Indian children under five: IHDS 2004/2005–2011/2012.

Decomposition	HAZ	%	WAZ	%
2011/2012	−1.1682 ***		−0.9484 ***	
	(0.035)		(0.030)	
2004/2005	−1.3931 ***		−1.0870 ***	
	(0.038)		(0.031)	
Total difference	0.2249 ***		0.1385 ***	
	(0.052)		(0.043)	
Explained	0.0195	9	0.0525 **	38
	(0.027)		(0.024)	
Unexplained	0.2054 ***	91	0.0860 **	62
	(0.054)		(0.043)	
Explained part				
Mother’s decision-making autonomy	−0.0028	−1	−0.0011	−1
	(0.005)		(0.003)	
Mother’s mobility autonomy	0.0099 ***	4	0.0061 **	4
	(0.004)		(0.002)	
Mother’s characteristics	0.0033	1	0.0161 **	12
	(0.007)		(0.007)	
Economic situation	0.0475 ***	21	0.0345 ***	25
	(0.017)		(0.013)	
Hygiene	0.0089	4	0.0027	2
	(0.007)		(0.005)	
Northwest	−0.0024	−1	−0.0011	−1
	(0.003)		(0.001)	
North	−0.0300 ***	−13	0.0025	2
	(0.007)		(0.004)	
South	0.0183 ***	8	0.0026	2
	(0.007)		(0.004)	
Central	−0.0040	−2	−0.0027	−2
	(0.005)		(0.003)	
West	0.0014	1	−0.0009	−1
	(0.002)		(0.002)	
Others	−0.0304 *	−14	−0.0062	−4
	(0.016)		(0.017)	
*N*	6445		7634	

Note: The dependent variables are the z-scores of height-for-age (HAZ) and weight-for-age (WAZ). Groups in the explained part are mother’s characteristics (mother’s BMI; mother’s education), household economic situation (household wealth); hygiene (water source; flushing toilet; and hand washing); and others (child age; child gender; father's education; caste; religion; rural resident) and 5 regional binary variables. Standard errors are in parentheses. * *p* ≤ 0.05, ** *p* ≤ 0.01, *** *p* ≤ 0.001.

**Table 4:** RIFR decomposition of socio-economic differences in *HAZ* and *WAZ* among Indian children under five: IHDS 2004/2005–2011/2012.

**Panel A: HAZ**	**25th**	**%**	**50th**	**%**	**75th**	**%**
2011/2012	−2.5250 ***		−1.3954 ***		0.0343	
	(0.058)		(0.038)		(0.049)	
2004/2005	−2.9957 ***		−1.6054 ***		−0.0186	
	(0.044)		(0.047)		(0.046)	
Total difference	0.4707 ***		0.2101 ***		0.0529	
	(0.072)		(0.061)		(0.072)	
Explained	0.1179 ***	25	0.0778 ***	37	−0.0521	
	(0.036)		(0.027)		(0.033)	
Unexplained	0.3527 ***	75	0.1323 **	63	0.1050	
	(0.081)		(0.063)		(0.075)	
Explained part						
Mother’s decision-making autonomy	−0.0125 *	−3	−0.0041	−2		
	(0.007)		(0.006)			
Mother's mobility autonomy	0.0174 ***	4	0.0112 **	5		
	(0.006)		(0.005)			
Mother’s characteristics	0.0209 **	4	0.0118	6		
	(0.009)		(0.009)			
Economic situation	0.1156 ***	25	0.0807 ***	38		
	(0.025)		(0.020)			
Hygiene	0.0085	2	0.0054	3		
	(0.009)		(0.010)			
Northwest	0.0032	1	−0.0028	1		
	(0.004)		(0.004)			
North	−0.0292 ***	−6	−0.0290 ***	14		
	(0.009)		(0.008)			
South	0.0214 **	5	0.0320 ***	15		
	(0.009)		(0.007)			
Central	0.0108	2	−0.0012	−1		
	(0.007)		(0.006)			
West	−0.0008	0	0.0019	1		
	(0.002)		(0.002)			
Others	−0.0374 **	−7	−0.0280 *	−14		
	(0.015)		(0.015)			
N	6445		6445		6445	
**Panel B: WAZ**	**25th**	**%**	**50th**	**%**	**75th**	**%**
2011/2012	−2.0814 ***		−1.1336 ***		−0.1021 **	
	(0.031)		(0.026)		(0.040)	
2004/2005	−2.3938 ***		−1.2779 ***		−0.1362 ***	
	(0.031)		(0.041)		(0.041)	
Total difference	0.3123 ***		0.1444 ***		0.0341	
	(0.046)		(0.050)		(0.060)	
Explained	0.0696 ***	22	0.0883 ***	61	0.0173	
	(0.021)		(0.026)		(0.029)	
Unexplained	0.2428 ***	78	0.0561	39	0.0168	
	(0.050)		(0.054)		(0.061)	
Explained part						
Mother’s decision-making autonomy	−0.0038	−1	0.0028	2		
	(0.004)		(0.004)			
Mother's mobility autonomy	0.0025	1	0.0042 *	3		
	(0.002)		(0.002)			
Mother’s characteristics	0.0198***	6	0.0226 ***	16		
	(0.007)		(0.008)			
Economic situation	0.0500 ***	16	0.0722 ***	50		
	(0.015)		(0.015)			
Hygiene	0.0023	1	−0.0055	−4		
	(0.005)		(0.005)			
Northwest	0.0009	0	0.0011	1		
	(0.001)		(0.002)			
North	0.0062	2	−0.0019	−1		
	(0.004)		(0.005)			
South	0.0042	1	0.0140 ***	10		
	(0.004)		(0.005)			
Central	0.0016	1	−0.0127 ***	−9		
	(0.004)		(0.004)			
West	−0.0002	0	0.0052 *	4		
	(0.002)		(0.003)			
Others	−0.0138	−4	−0.0136	−9		
	(0.010)		(0.016)			
N	7634		7634		7634	

Note: The dependent variables are the z-scores of height-for-age (HAZ) and weight-for-age (WAZ). Groups in the explained part are mother’s characteristics (mother’s BMI; mother’s education), household economic situation (household wealth); hygiene (water source; flushing toilet; and hand washing); and others (child age; child gender; father's education; caste; religion; rural resident) and 5 regional binary variables. Standard errors are in parentheses. Standard errors are in parentheses. * *p* ≤ 0.05, ** *p* ≤ 0.01, *** *p* ≤ 0.001.

**Table 5:** Non-linear decomposition of socio-economic differences in stunting, underweight and CIAF among Indian children under five: IHDS 2004/2005–2011/2012.

Decomposition	Stunting	%	Underweight	%	CIAF	%
2011/2012	0.3577		0.2746		0.4938	
2004/2005	0.4207		0.3182		0.5793	
Total difference	−0.0630 ***		−0.0436 ***		−0.0855 ***	
Explained	−0.0074	12	−0.0033	8	−0.0340	40
Unexplained	−0.0556	88	−0.0403	92	−0.0515	60
Explained part						
Mother’s decision-making autonomy	0.0001	0	0.0013	−3	0.0004	0
	(0.001)		(0.001)		(0.001)	
Mother’s mobility autonomy	−0.0019 ***	3	−0.0010 **	2	−0.0015 *	2
	(0.001)		(0.000)		(0.001)	
Mother’s characteristics	−0.0022 **	3	−0.0021 **	5	−0.0061 ***	7
	(0.001)		(0.001)		(0.002)	
Economic situation	−0.0123 ***	20	−0.0106 ***	24	−0.0164 ***	19
	(0.004)		(0.003)		(0.004)	
Hygiene	−0.0021	3	0.0002	0	−0.0035 *	4
	(0.002)		(0.001)		(0.002)	
Northwest	0.0007	−1	0.00004	0	−0.0029 ***	3
	(0.001)		(0.000)		(0.001)	
North	0.0048 ***	−8	−0.0011	3	0.0030 *	−4
	(0.001)		(0.001)		(0.002)	
South	−0.0043 ***	7	−0.0013	3	−0.0092 ***	11
	(0.001)		(0.001)		(0.002)	
Central	−0.0007	1	−0.0002	1	0.0022 **	−3
	(0.001)		(0.001)		(0.001)	
West	−0.0001	0	0.0002	0	−0.0007 **	1
	(0.000)		(0.001)		(0.000)	
Others	0.0104 ***	−17	0.0113 ***	−26	0.0008	−1
	(0.002)		(0.002)		(0.002)	
Number of replications	1000		1000		1000	

Note: The dependent variable is a dummy for whether the respondent is suffering or has suffered from stunting, underweight, and/or anthropometric failure. Groups in the explained part are mother’s characteristics (mother’s BMI; mother’s education), household economic situation (household wealth); hygiene (water source; flushing toilet; and hand washing); and others (child age; child gender; father's education; caste; religion; rural resident) and 5 regional binary variables. Bootstrapped-adjusted errors are in parentheses. * *p* ≤ 0.05, ** *p* ≤ 0.01, *** *p* ≤ 0.001.

**Table 6:** Non-linear decomposition of socio-economic differences in severe stunting and severe underweight among Indian children under five: IHDS 2004/2005–2011/2012.

Decomposition	Severe Stunting	%	Severe Underweight	%
2011/2012	0.1777		0.1020	
2004/2005	0.2455		0.1312	
Total difference	−0.0678 ***		−0.0292 ***	
Explained	−0.0136	20	−0.0041	14
Unexplained	−0.0542	80	−0.0251	86
Explained part				
Mother’s decision-making autonomy	0.0010	−1	0.0012 **	−4
	(0.001)		(0.001)	
Mother’s mobility autonomy	−0.0021 ***	3	−0.0008 **	3
	(0.001)		(0.000)	
Mother’s characteristics	−0.0015 *	2	0.0001	0
	(0.001)		(0.001)	
Economic situation	−0.0150 ***	22	−0.0059 ***	20
	(0.003)		(0.002)	
Hygiene	0.0001	0	0.0003	1
	(0.002)		(0.001)	
Northwest	−0.0007	1	0.0001	0
	(0.001)		(0.000)	
North	0.0023 **	−3	−0.0025 **	9
	(0.001)		(0.001)	
South	−0.0024 *	4	−0.0006	2
	(0.001)		(0.001)	
Central	−0.0016	2	−0.0008 **	3
	(0.001)		(0.000)	
West	0.00006	0	0.0004	−1
	(0.000)		(0.000)	
Others	0.0062 ***	−9	0.0044 ***	−15
	(0.002)		(0.001)	
Number of replications	1000		1000	

Note: The dependent variable is a dummy for the respondent is suffering or has suffered from severe stunting and underweight. Groups in the explained part are mother’s characteristics (mother’s BMI; mother’s education), household economic situation (household wealth); hygiene (water source; flushing toilet; and hand washing); and others (child age; child gender; father's education; caste; religion; rural resident) and 5 regional binary variables. Standard errors are in parentheses. * *p* ≤ 0.05, ** *p* ≤ 0.01, *** *p* ≤ 0.001.

## Appendix A

**Table A1:** Anthropometric failure groups among children under five.

Group	Description	Wasting	Stunting	Underweight
A	No failure: Children whose height and weight are above the age-specific norm and do not suffer from any anthropometric failure	No	No	No
B	Wasting only: Children with acceptable weight and height for their age but who have subnormal weight- for- height	Yes	No	No
C	Wasting and underweight: Children with above norm heights but whose weight-for- age and weight-for-height are too low.	Yes	No	Yes
D	Wasting, stunting, and underweight: Children who suffer from anthropometric failure on all three measures	Yes	Yes	Yes
E	Stunting and underweight: Children with low weight-for-age and low height-for-age, but who have acceptable weight for their height	No	Yes	Yes
F	Stunting only: Children with low height-for-age but who have acceptable weight, both for their age and for their short height.	No	Yes	No
Y	Underweight only: Children who are only underweight.	No	No	Yes

Source: Based on Nandy et al. [45]. Note: The theoretical combination of wasting and stunting is not physically possible because a child cannot simultaneously experience wasting and stunting and not be underweight.

**Table A2:** Regional definitions: IHDS 2004/2005–2011/2012.

Code	North	Code	Central	Code	East	Code	South	Code	Northeast	Code	West
01	Jammu & Kashmir	22	Chhatishgarh	10	Bihar	28	Andhra Pradesh	11	Sikkim	08	Rajasthan
02	Himachal Pradesh	23	Madhya Pradesh	19	West Bengal	29	Karnataka	12	Arunachal Pradesh	30	Goa
03	Punjab			20	Jharkhand	32	Kerala	13	Nagaland	27	Maharashtra
04	Chandigarh			21	Orissa	33	Tamil Nadu	14	Manipur	25	Daman and Diu
05	Uttaranchal					34	Pondicherry	15	Mizoram	26	Dadra and Nagar Haveli
06	Haryana							16	Tripura	24	Gujarat
07	Delhi							17	Meghalaya		
09	Uttar Pradesh							18	Assam		

**Table A3:** Blinder-Oaxaca decomposition of socioeconomic differences in malnutrition among Indian children under 5: IHDS 2004/2005–2011/2012(mothers aged ≤ 25 vs. mothers aged > 25).

**HAZ**	**Mothers aged ≤ 25**	**%**	**Mothers aged > 25**	**%**
2012	−1.1545 ***		−1.1752 ***	
	(0.066)		(0.042)	
2005	−1.3165 ***		−1.4346 ***	
	(0.064)		(0.047)	
Total difference	0.1620 *		0.2595 ***	
	(0.091)		(0.063)	
Explained	0.0500	31	0.0346	13
	(0.056)		(0.031)	
Unexplained	0.1120	69	0.2248 ***	87
	(0.098)		(0.065)	
Explained part				
Mother’s decision-making autonomy	−0.0035	−2	−0.0022	−1
	(0.010)		(0.005)	
Mother’s mobility autonomy	0.0189 **	12	0.0051	2
	(0.009)		(0.003)	
Mother age	0.0038	2	−0.0007	0
	(0.006)		(0.002)	
Mother characteristics	0.0020	1	0.0058	2
	(0.012)		(0.009)	
Economic situation	−0.0003	0	0.0737 ***	28
	(0.026)		(0.021)	
Hygiene	−0.0018	−1	0.0143	6
	(0.010)		(0.009)	
Northwest	0.0099	6	−0.0058	−3
	(0.009)		(0.004)	
North	−0.0119	−7	−0.0301 ***	−13
	(0.014)		(0.009)	
South	0.0094	6	0.0163 **	7
	(0.014)		(0.007)	
Central	0.0137	8	−0.0061	−3
	(0.015)		(0.004)	
West	−0.0033	−2	0.0004	0
	(0.006)		(0.002)	
Others	0.0129	8	−0.0361 **	−14
	(0.039)		(0.016)	
*N*	2168		4270	
**WAZ**	**Mothers aged ≤ 25**		**Mothers aged > 25**	
2012	−0.7914 ***		−1.0208 ***	
	(0.059)		(0.035)	
2005	−0.9903 ***		−1.1421 ***	
	(0.053)		(0.038)	
Total difference	0.1988 **		0.1213 **	
	(0.080)		(0.051)	
Explained	0.1009 **	51	0.0459 *	38
	(0.049)		(0.027)	
Unexplained	0.0979	49	0.0753	62
	(0.082)		(0.050)	
Explained part				
Mother’s decision-making autonomy	−0.0035	−2	−0.0002	0
	(0.009)		(0.003)	
Mother’s mobility autonomy	0.0119 *	6	0.0035	3
	(0.006)		(0.002)	
Mother age	−0.0023	−1	0.0007	1
	(0.005)		(0.002)	
Mother characteristics	0.0031	2	0.0191 **	16
	(0.013)		(0.009)	
Economic situation	−0.0019	−1	0.0508 ***	42
	(0.021)		(0.016)	
Hygiene	0.0066	3	0.0004	0
	(0.006)		(0.007)	
Northwest	0.0013	1	−0.0002	0
	(0.004)		(0.002)	
North	0.0127	6	−0.0016	−1
	(0.009)		(0.004)	
South	−0.0017	−1	0.0037	3
	(0.010)		(0.003)	
Central	0.0073	4	−0.0037	−3
	(0.010)		(0.003)	
West	−0.0050	−3	0.0008	1
	(0.004)		(0.002)	
Others	0.0724 *	36	−0.0274	−23
	(0.038)		(0.019)	
*N*	2594		5034	

Note: The groups are the same as in Table 3. The dependent variables are the *z*-scores of height-for-age (*HAZ*) and weight-for-age (*WAZ*). Groups in the explained part are mother’s characteristics (mother’s BMI; mother’s education), household economic situation (household wealth); hygiene (water source; flushing toilet; and hand washing); and others (child age; child gender; father’s education; caste; religion; rural resident) and 5 regional binary variables. Standard errors are in parentheses. * *p* ≤ 0.05, ** *p* ≤ 0.01, *** *p* ≤ 0.001.
